# Comparing apples and oranges (and pinecones): understanding patterns of reproductive disparity across seed plants

**DOI:** 10.1098/rspb.2026.1491

**Published:** 2026-10-07

**Authors:** Marco D'Ario, Andrew Leslie

**Affiliations:** Earth and Planetary Sciences, Stanford University, Stanford CA 94305, USA

**Keywords:** morphospace, discrete character, seed plants, reproductive biology

## Abstract

Seed plant reproductive structures are highly diverse, but analysing morphological disparity across the group is challenging because taxa are difficult to directly compare. Here, we develop an approach that accommodates divergent structures and unclear homologies, treating basic structural units as analogous and analysing characters within these units. We scored 284 acrogymnosperm and angiosperm cones and flower-fruits for 47 discrete characters and calculated morphological distances based on character mismatches among organ sets, treating character absence as a state. We then performed two analyses: one focused on the diversity of structural units and another focused on character diversity, where information from all structural units was collapsed into a single space. In both analyses, we found low reproductive disparity within most acrogymnosperm lineages but high disparity across them. Major angiosperm clades, by contrast, consistently show high disparity and occupy broad, overlapping regions of morphospace. Seed plant groups therefore appear to evolve disparity in fundamentally different ways: acrogymnosperms exhibit high levels among major lineages while angiosperms show high disparity both within and among major lineages. The approach we use to identify these patterns is also generalizable to other systems with serial homology and/or highly divergent morphologies.

## 1. Introduction

Seed plants exhibit a tremendous variety of reproductive structures, with those of angiosperms in particular having long been a focus of research into the evolution of phenotypic diversity (e.g. [1–7]). Quantifying this diversity has often centred on measuring continuously varying traits, ranging from studies of single features (e.g. seed mass or embryo size; [8,9]) to complex geometries (e.g. [10,11]). Many aspects of reproductive diversity, however, cannot be easily encapsulated by such variables; for example, it is difficult to assess differences in the number and functional interactions among floral organs through linear measurements alone. The use of discrete characters, which encode categorical or nominal features of organisms, has commonly been employed to analyse such aspects of morphology, especially by palaeontologists exploring disparity (morphological diversity) patterns across broad taxonomic groups and over long timescales (e.g. [12–15]).

Efforts to develop discrete character morphospaces, multi-dimensional mathematical spaces in which to analyse disparity patterns [16–18], are less common in botany than zoology but nonetheless have a long history [19–22]. Recent studies have particularly focused on angiosperms, combining fossils, new characters and molecular phylogenetics to analyse floral and seed disparity patterns at a variety of scales (e.g. [17,23,24]). Such studies have been able to characterize specific sets of features responsible for generating high disparity [25,26] as well as identify broad patterns across clades; for example, angiosperms appear to achieve high floral disparity early in their history [17,23]. This pattern is similar to that observed in many analyses of animals ([12]; although see [15]) and is consistent with the observation that morphological diversity and taxonomic richness are often decoupled over a clade's history [27–30].

For all their species richness and phenotypic diversity, however, angiosperms are only one lineage; broadly understanding seed plant disparity patterns requires analysing other groups as well. Living non-angiosperm seed plants form a clade called the Acrogymnospermae [31], which includes around 1000 species of conifers, cycads, *Ginkgo* and Gnetales (possibly nested inside conifers as traditionally defined; [32]), and the palaeobotanical record preserves many extinct Palaeozoic and Mesozoic non-angiosperm seed plant lineages (i.e. gymnosperms in the broad sense; see [33]). Qualitatively, gymnosperm reproductive structures appear less diverse than those of angiosperms, for example, living conifer and cycad cones do not exhibit the extreme variation in floral form, colour and organ arrangement that is thought to have arisen in angiosperms as a response to diverse pollination syndromes (e.g. [2,34–36]). Lower morphological diversity among gymnosperms also suggests that seed plant disparity in general underwent a substantial increase late in their history, as angiosperm fossils appear approximately 230 Myr after the first seeds [37] and extant acrogymnosperm lineages diverged from each other at least 70–160 Myr before angiosperms enter the record (and probably more; see [38,39]).

Quantifying whether gymnosperms really do occupy a restricted range of morphologies compared with angiosperms is challenging, however, because seed plant reproductive structures are difficult to directly compare. Acrogymnosperm cones and angiosperm flowers are composed of different numbers and kinds of organs, many with unclear homologies [40–42]. Even within angiosperms, where flowers tend to share a similar basic organography [1,43,44], clades often show deviations (e.g. independent origins of various staminode and petaloid organs). Such variation constitutes a major challenge when attempting to analyse reproductive disparity at a broad scale: which organs and structures should be used to calculate morphological distances, and what would such distances mean if organs are not shared across taxa? Any analytical framework must therefore accommodate both uncertainty in the evolutionary origins of basic organs and the frequent absence of particular organs among different groups [24].

In this study, we develop one approach to address these issues. We use a set of discrete morphological characters to separately score the basic structural units (e.g. floral organs) that compose a reproductive structure, treating these structures as analogous rather than homologous. We concatenate scorings from the various components and analyse morphological characters in a nested fashion within each component. This approach allows us to treat the absence of a given component as a character state rather than as non-applicable (see discussions in [15,45,46]), enabling us to calculate morphological distances between reproductive structures composed of different types of organs. We then perform two sets of analyses: one focused on differences in the number and type of basic structural units among taxa (the ‘total’ analysis) and one focused on character differences (the ‘union’ analysis, where information from all structural units in a taxon is collapsed into a single scoring). Using these approaches, we ask how reproductive disparity patterns among acrogymnosperms and angiosperms differ, particularly whether cones of major acrogymnosperm lineages are less diverse than the flowers and fruits of major angiosperm lineages.

## 2. Material and methods

### (a) Character scoring

We analysed reproductive structures from 88 acrogymnosperm and 196 angiosperm species, including representatives from nearly all extant acrogymnosperm genera. For angiosperms, taxon sampling provides a mix of close and distant relatives within several major groups/clades (e.g. ANN-grade lineages, magnoliids, monocots and eudicots). We typically sampled one genus per family to achieve wide phylogenetic coverage across major angiosperm lineages, although for some families we sampled multiple genera to capture more closely related taxa. Sampled angiosperms were based on a core set of taxa that we used in previous analyses of seed plant reproductive structures [47,48], supplemented with additional taxa chosen largely at random based on availability of morphological data and presence in the phylogeny used for analyses [49]. Morphological data are derived from published literature and personal observations, and all characters were scored for all taxa.

We scored reproductive structures for 47 discrete morphological characters (16 binary, 31 multi-state) that describe the shape, arrangement and physical interactions among the basic components that comprise reproductive structures. We term these basic components ‘structural units’, which generally correspond to traditional ‘floral organs’ like carpels, petals or cone scales. Specific characters describe the arrangement and architecture of these structural units, such as the extent to which they are fused or in physical contact with other units, whether they are fleshy at maturity, and the number of times a given unit type is reiterated within the reproductive structure (figure 1). Additional characters describe structural unit morphological features, such as the presence of specialized pollen reception (e.g. angiosperm stigmas) elements, scale-like elements (e.g. bracts, petals or cone scales) and ring elements like floral tubes, cups or envelopes (e.g. calyces or carpels). These morphological elements may constitute either the entire structural unit or represent components of a larger structure, such as free petal lobes (scale elements) attached to a corolla tube (a ring element). We also include several characters describing the morphology of sporangia (either pollen sacs or the seed nucellus) that may be borne on structural units. Some of these characters are dependent on others, for example, ‘scale’ and ‘ring’ elements include seven and eight sub-characters, respectively, that cannot be scored if these elements are absent. We therefore organized some characters into nested sets for subsequent analyses (see below); in total, we grouped the 47 characters into 19 nested sets listed in figure 1.

**Figure 1.**
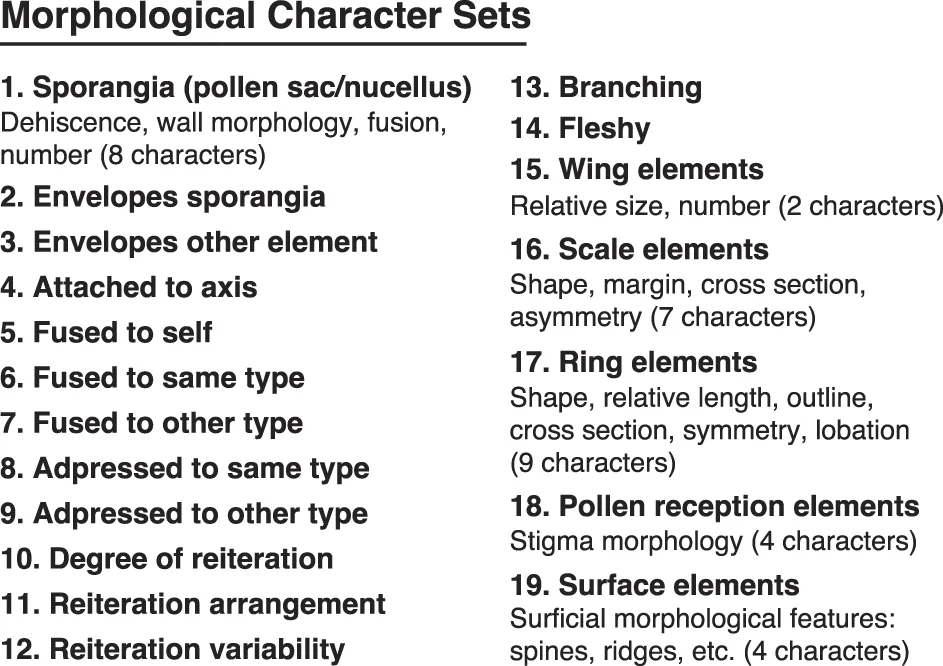
Morphological characters used in this study. Characters describe the basic morphology, arrangement and physical interactions shown by each type of structural unit (i.e. floral organ) that comprises a given seed plant reproductive structure. We organized these 47 characters into 19 nested sets for distance calculations; not every character contains nested sub-characters. For a full list of characters, see supplementary material.

Seed plant reproductive structures change form over their ontogeny as they perform different functions in pollination, seed protection and seed dispersal. We generally scored structural units based on their morphology at developmental maturity, except for characters relating to pollen reception elements (character set 18), which we scored at pollination. The timing of maturity varies among structural units, however; flower stamens, petals and sepals typically mature and function at pollination while carpels continue to develop as fruits. Scorings then reflect a combination of mature forms for each floral organ in a taxon, creating a morphological summary over ontogeny (see similar approach in [47,48]). For example, in a typical angiosperm we score floral bracts, perianth organs, staminal organs and carpel stigmatic surfaces based on their morphology at pollination, but we score other aspects of carpel morphology at fruit morphology; we refer to the resulting combination as ‘flower-fruit’ throughout the text. For further details relating to our characters and scoring approach, see supplementary notes in the supplementary material).

### (b) Structural unit scoring

Vascular plant reproductive structures typically consist of multiple kinds of structural units with different morphologies and functions. For a given reproductive structure, we scored each type of structural unit separately for the characters described above and concatenated them into a single character block. Homologies among different kinds of structural units are often unclear, however; so to compare taxa across groups we assign structural units to a set of analogous categories (figure 2a). We divide reproductive structures into integuments (I), including the seed coat (I.1) and arils if present (I.2), first-order axes (A1; flower stalks or simple cone axes) that bear first-order lateral units (L1) and second-order axes (A2; inflorescence or equivalent axes) that bear second-order lateral units (L2A). Third or higher-order axes may also be present but are treated as additional A2 units. L2B units refer to bracts subtending the entire inflorescence.

**Figure 2.**
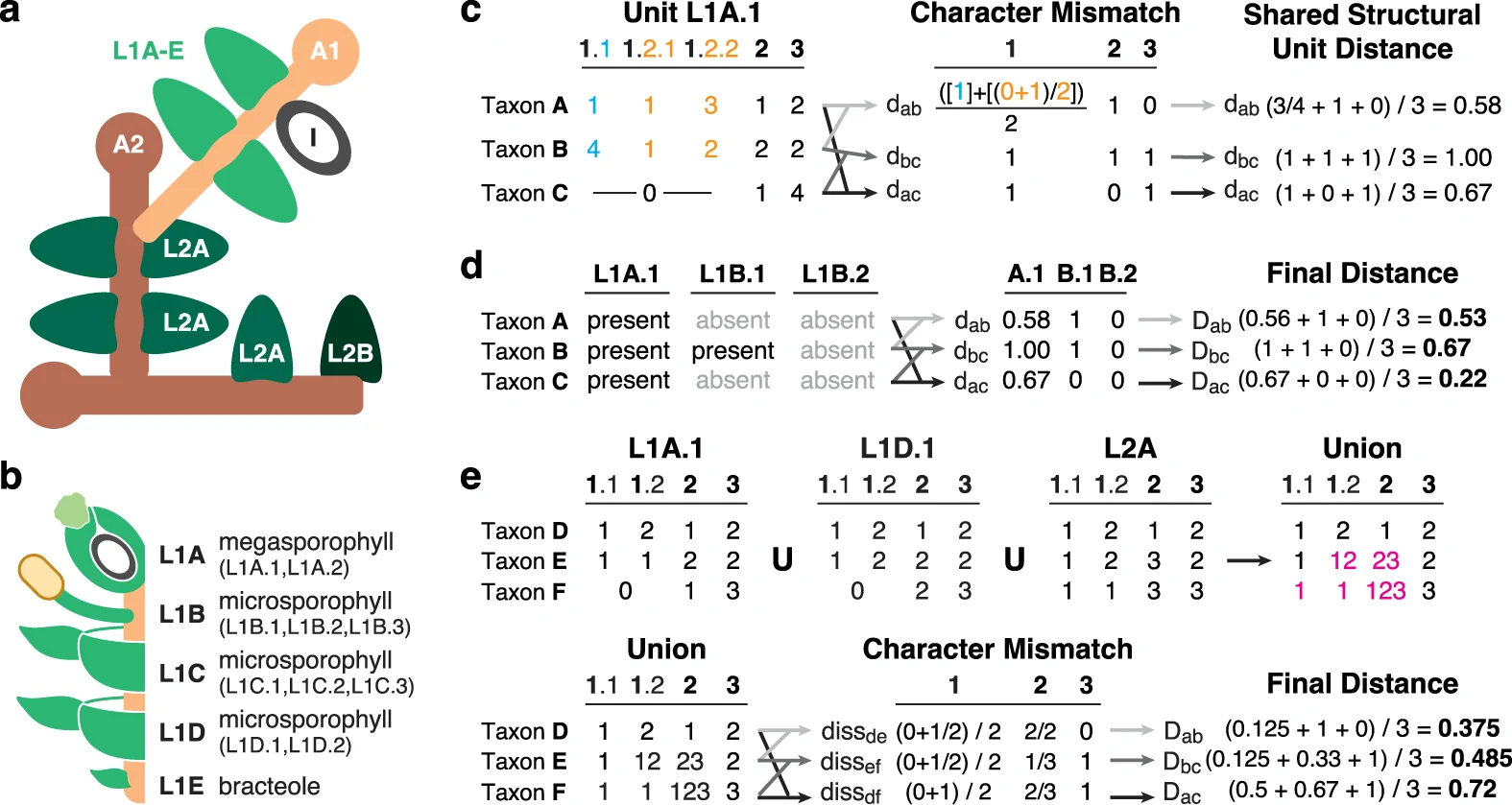
Structural unit categorization and example morphological distance calculations. (a) Example reproductive shoot system with first-order axes (A1) bearing integumentary (I) units and various lateral units (L1A–E). Higher-order axes (A2) also bear lateral units (L2A/B), where L2B subtend entire system. (b) Major categories of L1 structural units; L1A–D include multiple subtypes. (c) Within-structural unit pairwise distance calculations for three taxa with one representative unit type (L1A.1) described by one nested character set (1.1–1.2.2) and two non-nested characters ′2′ and ′3′. The nested character set consists of three sub-characters, two of which are further nested (orange). To compute distances, mismatches among the most nested characters are tallied first and averaged before repeating with mismatches at the next nested level. Non-applicable characters are scored as absent (0) and treated as character mismatches. Pairwise distances among taxa for a shared structural unit are then found by averaging mismatches among nested and non-nested characters. (d) Final pairwise distances among the same taxa based on three types of units. Distances for shared the shared L1A.1 unit are from (c). Distances for L1B.1, found only in taxon B, are maximum morphological distances ( = 1) while those for L1B.2, absent in all taxa, are minimum distances ( = 0). Distances in panels (c) and (d) correspond to the ‘total’ analysis discussed in the text. (e) Pairwise distance calculation in the ‘union’ analysis for three different hypothetical taxa, where character states from three representative units (L1A.1, L1D.1 and L2A) are combined into multi-state characters (pink) if different. Distances are then calculated as before except that character mismatches are based on fraction of digit mismatches among characters.

We then assign lateral units to additional categories based on functionality and position (figure 2a). L1A and L1B units bear ovules and pollen sacs, respectively, with sterile pistillodes and staminodes categorized as different types of L1A and L1B units. L1C, L1D and L1E categories accommodate various sterile appendages, including general ‘perianth’ (L1D; tepals, sepals or bracts), additional differentiated ‘perianth’ distal from L1D units (L1C; petals or petaloid organs) and laminar organs proximal to L1D units (L1E; bracteoles/prophylls). These categories may be further differentiated, for example, Fabaceae flowers develop distinct keel, wing and banner petals that we treat as LIC subtypes (LIC.1,2,3). In total, we assigned each structural unit in a given cone or flower/fruit to one of 18 possible architectural/functional categories (I[1,2], A1, A2, L1A[1,2], L1B[1,2,3], L1C[1,2,3], L1D[1,2], LIE, L2A[1,2], L2B). No single taxon expresses all categories and most taxa lack most of them; absences accounted for approximately 60% of the cells in a matrix composed only of structural unit presence/absences. See supplementary material for a detailed description of our categorization approach.

### (c) Calculating morphological distances

We calculated morphological distances between taxon pairs using aligned sets of categorized structural units (figure 2c,d). In general, we calculated distances in a nested fashion using character state mismatches (Hamming distances) among corresponding characters: distances at the lowest level were computed as mismatches among the most nested character subsets, which were then averaged to compute distances at higher levels (figure 2c). If an individual character or nested character set was absent in a taxon, it was treated as exhibiting maximum distance (= 1.0) with taxa that did produce them. Morphological distances between structural units shared by pairs of taxa were then given as the sum of character mismatch distances divided by the total number of characters (figure 2c). Structural units present in only one taxon of a pair were treated as exhibiting maximum distance ( = 1.0) while those absent in both taxa were considered to represent zero morphological distance (figure 2d). Total pairwise distance between taxa is the sum of shared present, shared absent and non-shared structural unit distances divided by the total number of structural unit categories (figure 2d). We refer to use this distance metric as the ‘total’ analysis throughout the paper.

Structural unit categorization will inevitably affect specific distance values because it determines which units are shared or absent among taxa. To generate distances defined only by characters and not unit categories, we performed a second analysis where we collapsed corresponding characters from all structural unit types into a single set. This ‘union’ analysis combines variable characters across multiple organs into new states, for example, a character scored as state ‘1’ in one type of structural unit and ‘2’ in another type would score as ‘12’ (figure 2e). To calculate distances among taxa in this space, we used the same basic approach as described above but modified so that the extent of character mismatch reflects the number of shared digits. For example, states ‘1’ and ‘1234’ would have a dissimilarity of 3/4 because they share one digit (‘1’) of four (‘1234’). Taxa that produce more, and more varied, organs will generally show longer numerical strings for individual characters and thus larger distances from taxa that produce fewer or less morphologically variable organs.

Many plants produce separate staminate (pollen-producing) and ovulate (seed-producing) reproductive structures. In these cases, we calculated morphological distances for both types separately, although we primarily discuss seed-producing structures because they are the most diverse. ‘Seed-producing’ here refers to any structure that produces viable seeds, including ovulate acrogymnosperm cones, pistillate angiosperm flowers and bisexual angiosperm flowers. By contrast, ‘pollen-producing’ refers to those that produce pollen only, including staminate angiosperm flowers and acrogymnosperm pollen cones. Because comparing bisexual with unisexual flowers or cones could introduce biases (e.g. unisexual structures lack L1B units by definition), we also amalgamated structural units from unisexual pollen- and seed-producing cones or flowers into a single hypothetical ‘combined’ structure. Here, we used the approach described for the union analysis, but instead of combining columns (characters) we combined rows (pollen-producing and seed-producing structures from a single species). In most cases, this results in adding an L1B unit to the seed-producing structure.

Lastly, distance calculations in this study differ from those in many discrete character analyses. To explore potential biases of this approach, we also calculated distances using a more traditional or ‘classical’ metric. In this case, we eliminated nested structure by calculating distances simply as state mismatches among all characters equally rather than averaging mismatches among nested sets. The ‘classical’ metric also treats absent characters as non-applicable and removes them from pairwise distance calculations by using only characters/structural units shared between pairs of taxa.

### (d) Analysing morphological distances

We primarily analysed disparity patterns by comparing distributions of pairwise morphological distances among taxa and lineages, using raw distances derived from the metrics described above. We also compared pairwise morphological distances among taxa against patristic distances, in this case, estimated divergence ages from molecular phylogenies [49,50]. For angiosperms, we primarily discuss results from the ‘young’ rather than the ‘old’ dated tree in [49] because the former was calibrated to better align with the fossil record. For species not sampled in [49], we used dates from congeners (the tree is sampled at the genus level and thus provides the same divergence dates).

We visualized reproductive morphospaces with principal coordinates (PCO) ordinations of pairwise distances using the ‘wcmdscale’ function in the R-package *vegan* [51], with a Lingoes correction applied to negative eigenvalues. We calculated associations between PCO axes and individual characters using *R*^2^ values based on linear models of PCO axis scores fit to original character values. We determined the relative ‘contribution’ of each character to a given PCO axis by dividing its *R*^2^ value by the sum of *R*^2^ values for all characters and multiplying by 100.

Morphospaces based on discrete character data are inherently discontinuous, but this feature will be especially pronounced in the total analysis because the way we define structural unit categories creates impossible combinations, for example, LIC units will only be assigned if L1D.1 units are already present and L1C.2 units cannot occur without L1C.1 units. To better understand the structure of the total analysis morphospace, we developed a hypothetical morphospace based on simulated structural unit combinations. Starting with 18 organ types, we treated the presence or absence of these types as binary characters and generated all 3284 possible combinations that result from our assignment rules (see supplementary material for list). We then rescored structural unit type presence/absence in our observed taxa as binary characters and combined this matrix with that of the simulated combinations. Using this matrix, we calculated total analysis morphological distances as described above and ordinated the results using PCO axes. These and all other analyses were performed in R [52]. Custom scripts for character unions, morphological distance calculations, PCO axis contributions and hypothetical structural unit combinations are available on Dryad [53].

## 3. Results

### (a) Total analysis

In the total analysis, which incorporates separate character information from each type of structural unit, seed-producing cones within acrogymnosperm clades other than Gnetales exhibit small pairwise distances (figure 3a). By contrast, distances are consistently greater among seed-producing flower-fruits within major angiosperm grades or clades (figure 3a); median values among early-diverging (ANA-grade taxa, Chloranthaceae and *Ceratophyllum*), magnoliid, monocot and eudicot taxa average more than seven times those of non-Gnetalean acrogymnosperms (figure 3a). Pairwise morphological distances within each major angiosperm analysis group/clade are similar to those of angiosperms in aggregate, whereas those of most acrogymnosperm clades are much smaller than total acrogymnosperm distances (figure 3a). High reproductive disparity therefore appears to be a general feature of major angiosperm clades but emerges among acrogymnosperms only across deep phylogenetic splits.

**Figure 3.**
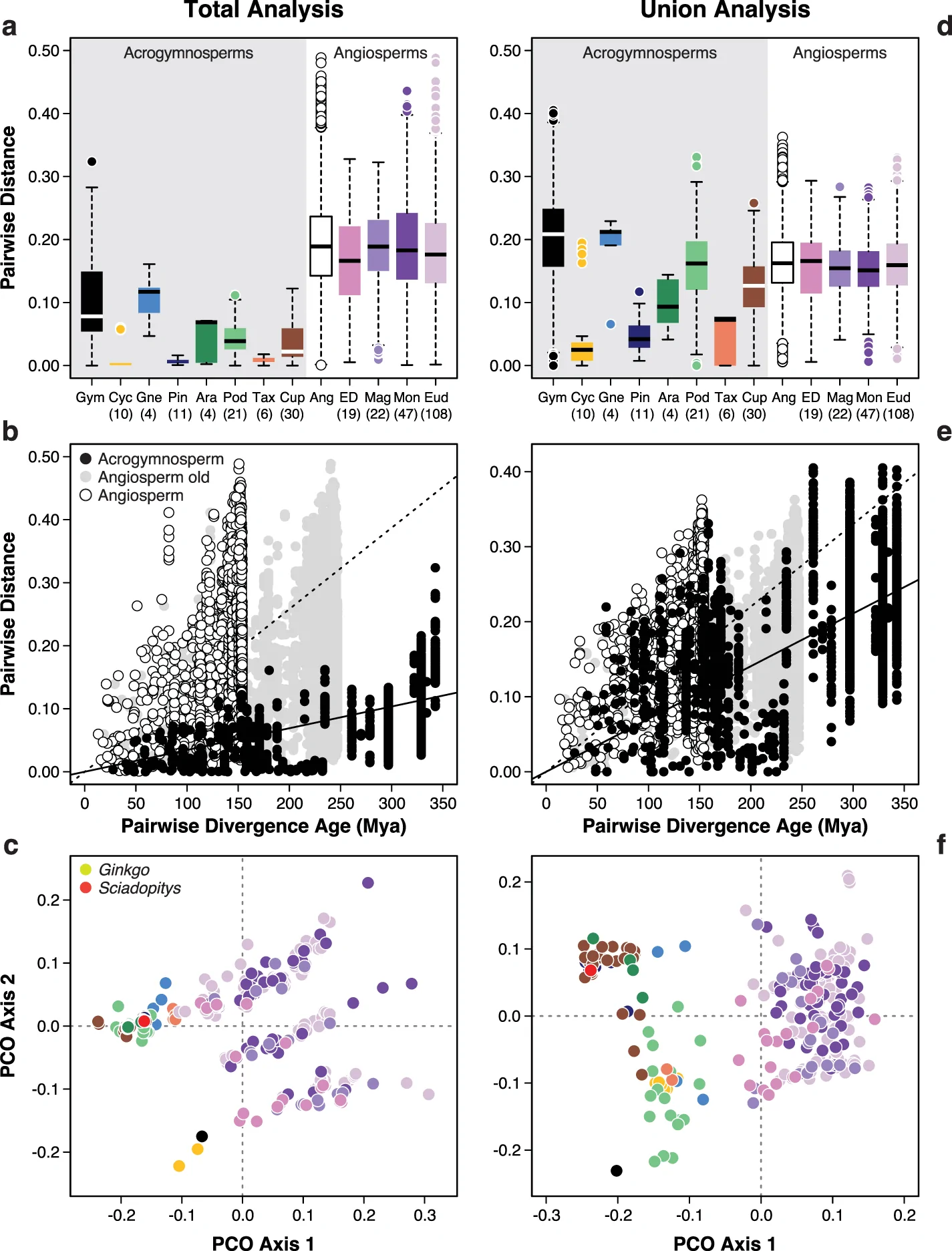
Pairwise morphological distances and morphospace occupation patterns for seed-producing structures (ovulate cones, pistillate flower-fruits and bisexual flower-fruits). (a,d) Within-group distances for acrogymnosperms and angiosperms in the total and union analyses. Boxplots show median (thick line), interquartile range (boxes), 1.5 times interquartile range (whiskers) and outliers. Numbers below group names indicate number of taxa in each group. (b,e) Pairwise distances versus estimated pairwise divergence ages for acrogymnosperm and angiosperm taxa in the total and union analyses. Two angiosperm ages represent alternative age calibrations; wefavour younger ages because they better align with the fossil record. Lines indicate best fit regression slopes for acrogymnosperms (solid) and angiosperms (dashed; using younger ages). (c,f) PCO axes 1 and 2 of pairwise distances from the total and union analyses. Colours correspond to groups in panels (a) and (d) plus two monotypic acrogymnosperms (*Ginkgo, Sciadopitys*). Ang = all angiosperms, Ara = Araucariaceae, Cup = Cupressaceae, Cyc = cycads, ED = ‘early-diverging’ angiosperms including ANA-grade, *Ceratophyllum*, and chloranthaceae, Eud = eudicots, Gne = Gnetales, Gym = all gymnosperms, Mag = magnoliids, Mon = monocots, Pin = Pinaceae, Pod = Podocarpaceae, Tax = Taxaceae.

Comparing pairwise taxon morphological distances against patristic distances supports this interpretation, as angiosperm flower-fruits show greater morphological distances at younger divergence ages in the total analysis. The regression slope in angiosperms is more than three times that of acrogymnosperms (0.00064 and 0.00017, respectively, for linear models with intercepts fixed at zero; figure 3b). The high angiosperm slope is not owing to a single clade but occurs in all groups analysed here (supplementary material, figure S1). By contrast, large pairwise distances among acrogymnosperms in the total analysis primarily occur across splits corresponding to family- or order-level comparisons (supplementary material, figure S1).

PCO ordinations are consistent with patterns described above for raw pairwise distances; acrogymnosperm seed cones occupy more restricted and localized regions of morphospace than angiosperm flower-fruits (figure 3c). The first two PCO axes explain around 55% of the variation in the original data (based on positive eigenvalues prior to Lingoes correction). More expansive angiosperm morphospace occupation is primarily owing to variation on axis 1, which reflects more diverse first-order lateral units (e.g. stamens, staminodes, petals and sepals); PCO 1 is most strongly associated with characters belonging to megasporophyll, microsporophyll and perianth units (supplementary material, figure S2). By contrast, clustering of both acrogymnosperm and angiosperm taxa on axis 2 is owing to similar combinations of second-order units (e.g. A2, L2A and L2B; supplementary material, figure S2). Low scores correspond to simple seed cones (e.g. cycads) or solitary flower-fruits without inflorescence organs, intermediate scores correspond to inflorescences lacking developed bracts and high scores with compound seed cones (e.g. most conifers) or inflorescences with well-developed bracts.

Total analysis disparity patterns therefore appear to be primarily determined by the presence/absence of structural unit types rather than specific characters within units. In fact, if character data are eliminated altogether and structural units simply scored as binary presence/absence, the resulting distance matrix and PCO ordination are very similar to those of the full character total analysis (supplementary material, figure S3). A hypothetical morphospace of structural unit presence/absence further supports this idea (supplementary material, figure S4). Here, both real and simulated structural unit combinations plot continuously along the new PCO axis 1, which like the empirical total analysis PCO axis 1 is correlated with the diversity of ‘perianth’-type L1 units. The next three axes in this hypothetical morphospace circumscribe highly discontinuous spaces, however, with occupiable regions dictated by the integer number of staminal units (microsporophylls, stamens and/or staminodes; PCO axis 2), second order units (inflorescence axes and bracts; PCO axis 3) and megasporophyll units (carpels and pistillodes; PCO axis 4).

### (b) Union analysis

In the union analysis, which emphasizes character diversity rather than structural unit diversity, acrogymnosperm seed-producing cones show greater within-clade pairwise distances, sometimes reaching angiosperm levels (figure 3d). Especially high pairwise distances within Gnetales and Podocarpaceae reflect highly variable seed cone morphologies in these groups; both produce fleshy ‘berry’ and ‘drupe’-like structures at maturity as well as dry sclerified cones, and both have evolved different types of envelopes (scored as ‘ring’ elements) that cover seeds throughout their ontogeny. Acrogymnosperm pairwise distances in the union analysis also increase more rapidly with divergence age than in the total analysis, although still less than those among angiosperm flower-fruits (figure 3e; slope = 0.0003 and 0.0005, respectively). The higher slope among acrogymnosperms in the union analysis is primarily driven by Cupressaceae and Podocarpaceae; morphological distances within cycads, Pinaceae and Taxaceae exhibit little increase with divergence age (supplementary material, figure S1). In general, disparity among acrogymnosperms in the union analysis is highly variable among clades, while angiosperm groups analysed all show large pairwise distances (figure 3d).

Consistent with patterns in the raw distances, acrogymnosperm morphospace occupation increases substantially in the union analysis (figure 3f). The union analysis ordination space is primarily defined by a suite of characteristic angiosperm traits on PCO axis 1, including postgenital fusion (in the carpels of many derived angiosperms) and diverse pollen reception morphologies expressed at pollination on the carpel, and on PCO axis 2 by seed dispersal traits expressed in carpels or cone scales at maturity, especially the presence of fleshy tissues and seed/fruit wings (supplementary material, table S1). Clustering by lineage remains strong among acrogymnosperms in the union morphospace, however, because the ‘fruit’ and seed traits associated with PCO axis 2 are often lineage-specific, separating clades with woody seed cones at maturity (e.g. Araucariaceae and Pinaceae) from those that typically make fleshy cones (e.g. Podocarpaceae and Taxaceae).

### (c) Comparison with other distance metrics

We repeated total and union analyses using an alternative method of calculating pairwise distances where characters were not treated as nested sets, and absent characters/structural units were treated as non-applicable (the ‘classical’ metric, as mentioned previously). Acrogymnosperm disparity in the total analysis using this metric was relatively high (figure 4a,b) because ‘classical’ distances are calculated only from structural units shared by pairs of taxa; by contrast, they were smaller in the original total analysis because acrogymnosperms typically produce few kinds of structural units and thus taxa share many absences (= 0 distance). The ‘classical’ version of the union analysis (supplementary material, figure S5) is more similar to our initial analysis because the union process eliminates structural unit absences to begin with. Differences are then primarily owing to the use of nested character sets in the initial analysis, which effectively downweights characters that occur within them. The distance metric introduced in this study therefore de-emphasizes certain aspects of morphological variation, although the ‘classical’ approach is also biased here by overcounting dependent characters. Despite these differences, the most basic disparity pattern across acrogymnosperm and angiosperm seed-producing cones and flower-fruits is consistent across analyses; namely, that individual acrogymnosperm lineages tend to show lower disparity and occupy more restricted regions of morphospace than angiosperm lineages.

**Figure 4.**
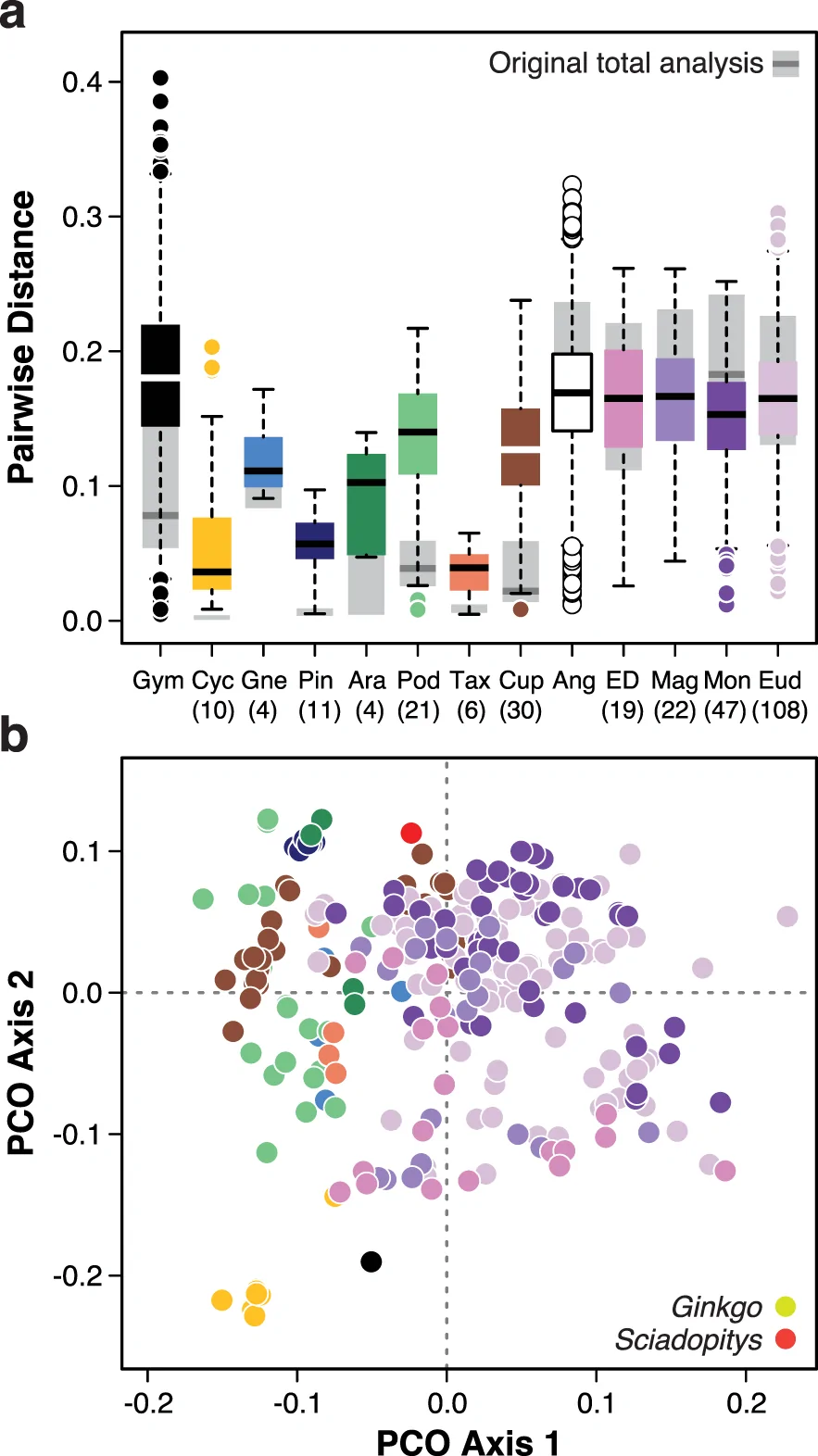
Comparison between morphological distances from original total analysis of seed-producing structures and those based on the ‘classical’ metric. In the latter, characters were not nested for analysis and non-applicable characters were removed from distance calculations. (a) Within-group pairwise distances for acrogymnosperms and angiosperms; boxplots show median (thick line), interquartile range (IQR; boxes), 1.5 times IQR (whiskers) and outliers. Grey boxes indicate median and IQR from the original total analysis. (b) PCO axes 1 and 2 based on ‘classical’ pairwise distances. Colours correspond to groups in panel (a) plus two monotypic acrogymnosperms (*Ginkgo*, *Sciadopitys*). Ang = all angiosperms, Ara = Araucariaceae, Cup = Cupressaceae, Cyc = cycads, ED = ‘early-diverging’ angiosperms including ANA-grade, *ceratophyllum*, and chloranthaceae, Eud = eudicots, Gne = Gnetales, Gym = all gymnosperms, Mag = magnoliids, Mon = monocots, Pin = Pinaceae, Pod = Podocarpaceae, Tax = Taxaceae.

### (d) Pollen-producing and combined structures

Pollen-producing structures (e.g. staminate cones or flowers) show broadly similar patterns to seed-producing structures: low disparity among non-Gnetales acrogymnosperms relative to angiosperm groups and a slower increase in pairwise morphological distance with patristic distance among acrogymnosperms compared with angiosperms (supplementary material, figure S6). Likewise, when structural units from both pollen-producing and seed-producing cones or flower-fruits are combined into single hypothetical construct that encapsulates the entire reproductive morphology of a taxon, disparity patterns are generally similar to those of seed-producing structures (supplementary material, figure S7).

## 4. Discussion

### (a) Acrogymnosperm and angiosperm disparity patterns

Characters in this study are meant to capture the basic morphology and organization of seed plant reproductive structures, including the different types of parts that comprise them, the diversity of shapes expressed as these parts mature, and how parts physically interact to form intricate flowers/cones at pollination or compact fruits/cones at maturity. In terms of these structural and functional aspects, the most consistent result of our analyses is that acrogymnosperm and angiosperm lineages evolve reproductive disparity in different ways. Acrogymnosperms generally show low disparity within major clades but may show high disparity among them. Or put another way, acrogymnosperms often show similar ways of building cones within groups but different ways between groups. By contrast, large angiosperm clades like magnoliids, monocots and eudicots (all younger than acrogymnosperm families) each show comparably high disparity and occupy of broadly overlapping regions of character space owing to similar trends in floral evolution, such as the repeated evolution of differentiated perianth, syncarpy [54,55], floral element fusion [56,57] and basic fruit morphologies (e.g. [58,59]).

These patterns could reflect methodological biases, especially in the total analysis where morphological distances are largely determined by the number of structural unit types, and thus acrogymnosperms tend to show low disparity. But our approach does not force small pairwise distances upon acrogymnosperms, as Gnetales exhibit high disparity. Rather, the pattern highlights an important feature of acrogymnosperm reproductive structures: most simply do not exhibit the diversity of lateral organs characteristic of angiosperms (e.g. various staminodes, petals, sepals, etc.). Traditional approaches to analysing discrete characters, which would treat these floral organs as non-applicable for acrogymnosperms, therefore cannot fully capture this difference. Indeed, disparity patterns in the ‘classical’ total analysis are more similar to those of the union analysis where organ information has been collapsed. One advantage of the approach presented in this study is that these different aspects of disparity can be analysed in a single framework.

The disparity patterns recovered here are also consistent in important ways with those of previous studies focused on particular groups (e.g. [17,23–26,60]), suggesting that our analysis does capture realistic patterns. Specifically, major angiosperm clades in these studies typically occupy broad and overlapping regions of floral morphospace, with high disparity achieved early in clade history [17,23]. Likewise, major angiosperm clades equivalent to those used here (e.g. monocots and eudicots) show broad overlap in seed/fruit morphospace and greater morphological diversity than acrogymnosperms [24]. And as in our results, seed cone morphospace occupation among conifers has been found to be structured by major clade, with greater morphological diversity in Podocarpaceae and Cupressaceae [60].

Assuming, then, that our patterns reflect biological reality, pollination biology offers a potential explanation for the high disparity of angiosperms. This group often uses specialized lateral organs to attract animal pollinators and facilitate pollen transfer, functions that have long been thought to promote morphological diversity [34–36,61,62]. Biotic pollination is not unique to angiosperms, however; living cycads and many Gnetales are insect pollinated [63–65]. Biotic pollination syndromes are also not consistently associated with disparity among acrogymnosperms; some entomophilous Gnetales do produce complicated reproductive cones (e.g. *Ephedra foeminea* and *Welwitschia*) but cycads also form specialized interactions with pollinators [65] and show low disparity. Likewise, there is increasing evidence that insect pollination was widespread among Mesozoic gymnosperms [66], but complicated flower-like morphologies are rare in the fossil record [47,48]. Biotic pollination by itself therefore does not appear sufficient to explain differences in disparity between acrogymnosperms and angiosperms, although angiosperms are more likely to develop morphologically specialized reproductive structures to engage in these interactions.

Alternatively, seed plant disparity patterns may simply reflect low rates of evolution in acrogymnosperms compared with angiosperms, resulting in fewer major morphological shifts within lineages and slower exploration of morphological space, even in animal-pollinated lineages. Nucleotide substitution rates among acrogymnosperms are roughly seven times lower than in angiosperms [67], and acrogymnosperms show little genomic dynamism, with consistently large genome sizes and infrequent polyploidy [42,68,69]. By contrast, fast substitution rates, variable genome sizes and frequent polyploidy have all been implicated in angiosperm diversification and disparity [70–73]. It is notable in this regard that Gnetales also show high rates of molecular evolution, relatively small genomes and evidence of polyploidy [69,74,75]. Putative fossil Gnetales are also varied in their organography (e.g. [76,77]), suggesting high disparity has long characterized the group. Perhaps, then, high rates of molecular evolution and genomic dynamism underlie the evolution of seed plant reproductive disparity, particularly with regard to the recruitment and differentiation of lateral organs to perform specialized functions in pollination.

### (b) Future directions

Our results suggest that seed plant reproductive disparity should show pulsed increases through time, as large pairwise distances are associated with deep divergences among acrogymnosperm lineages in the late Palaeozoic [39] and the evolution of major angiosperm clades in the Early to Mid-Cretaceous. Such a pattern would mirror changes in reproductive complexity [47,48] and highlights the importance of angiosperm diversification in determining long-term patterns of reproductive evolution in plants. Pulsed rises in disparity can be directly tested by incorporating fossil reproductive structures into our analyses, although including them is not trivial owing to long-standing questions regarding homology among Mesozoic gymnosperm groups (e.g. [40–42]). The approach we develop here can readily accommodate different homology hypotheses—organs can be easily recategorized for multiple sets of analyses—but properly exploring their effects on disparity patterns requires a detailed treatment of alternative homology schemes that is beyond the scope of this study and is the focus of ongoing work.

In a more general sense, this study introduces an approach to calculating morphological distances among fundamentally dissimilar taxa by making use of characters that would generally be considered non-applicable. Other studies have developed techniques for incorporating non-applicable character data for phylogenetic [78] or disparity analyses, either by reweighting dependent characters [46] or by treating absence as a state [15,45], but our approach is tailored to the modular nature of plant reproductive structures: we score cone or floral organs as separate entities, concatenate them and then analyse their respective characters in a nested fashion to build morphological distances from multiple organ comparisons. This approach is potentially adaptable to a variety of systems and questions, for example, morphological or anatomical characters describing leaves and roots could be used to study vegetative disparity patterns across vascular plants while also incorporating different analogy/homology schemes [79–81]. At the other end of the taxonomic spectrum, this approach could be used to analyse disparity patterns within clades where homologies are well understood; here the structural unit categorization would simply reflect known homologies. In such cases, disparity differences could also be more readily interpreted in terms of phylogenetic comparative methods. And beyond plants, our approach is potentially relevant for any group that exhibits highly diverse morphologies with unclear homologies, as it provides a way to compare analogous organs and characters. Although such an approach necessarily limits the types of evolutionary inferences that can be drawn from the results, it nevertheless provides a way to quantify morphological variation across widely divergent groups.

## Supplementary Material



## Data Availability

All data and scripts used in this study are available on Dryad [53]. Supplementary material is available online [82].
