# Supplementary material for: Comparing apples and oranges (and pinecones): understanding patterns of reproductive disparity across seed plants
Source: Proc Biol Sci. 2026 Oct 7;293(2080):20261491. doi: 10.1098/rspb.2026.1491 (PMC13686004; doi:10.1098/rspb.2026.1491)
Supplement: Supplementary file 1 [file rspb.2026.1491.zip › rspb.2026.1491/rspb20261491_si_001.pdf]

Electronic Supplementary Material

Title: Comparing apples and oranges (and pinecones): understanding patterns of reproductive disparity across seed plants

Authors: Marco D'Ario and Andrew B. Leslie

Journal: Proceedings of the Royal Society B: Biological Sciences

DOI: 10.1098/rspb.2026.1491

Supplementary Figures and Tables

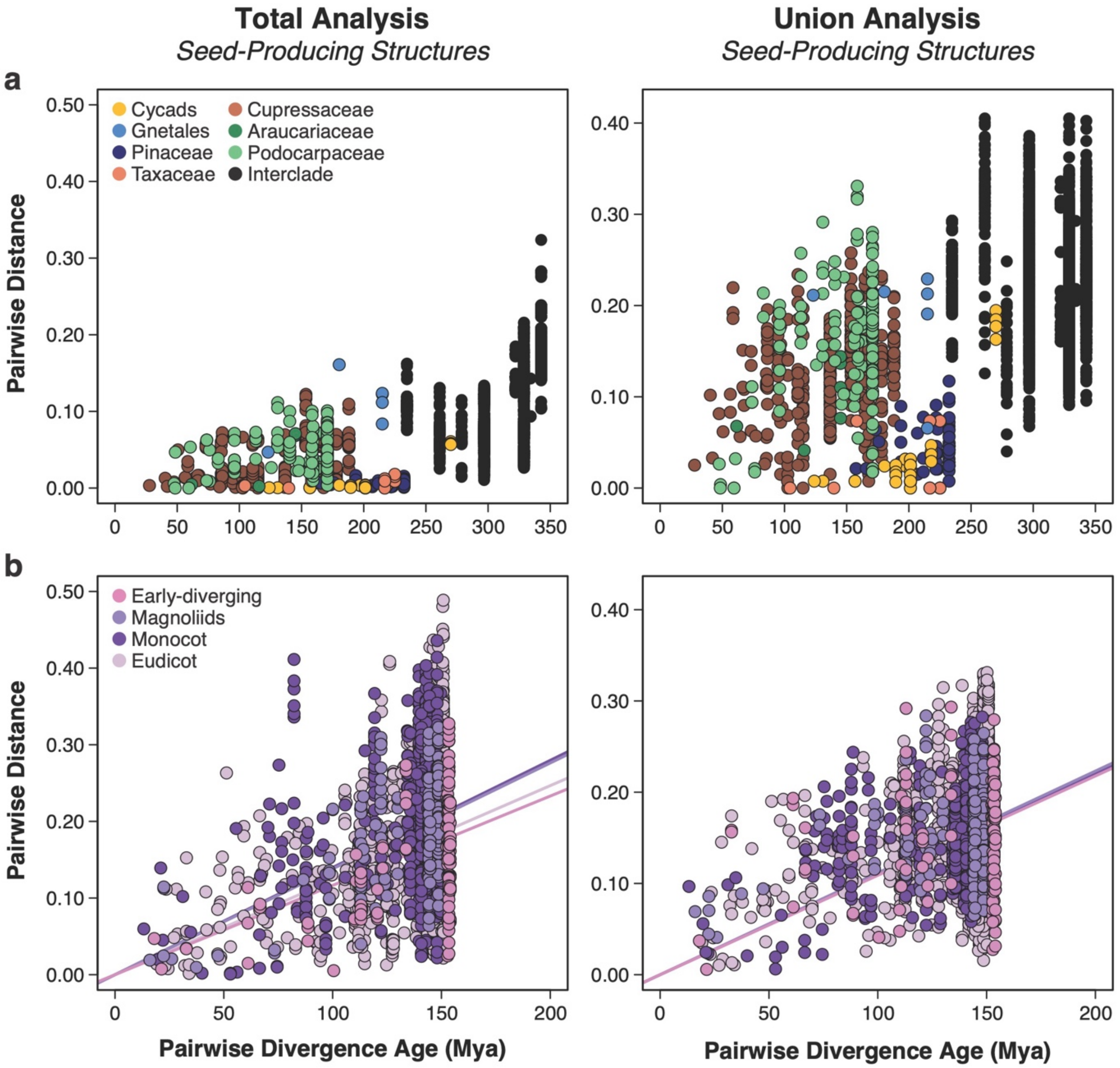

Figure S1. Morphological pairwise distances from the total and union analyses versus pairwise divergence ages for seed-producing reproductive structures by group/clade. (a) Gymnosperm clades; colored symbols indicate intraclade species pairs while black 'Interclade' points correspond to species comparisons between families or orders. (b) Angiosperm clades; interclade comparisons not shown. Lines show best-fit regression slopes for groups. Divergence ages derived from Stull et al. (2021) and Zuntini et al. (2024).

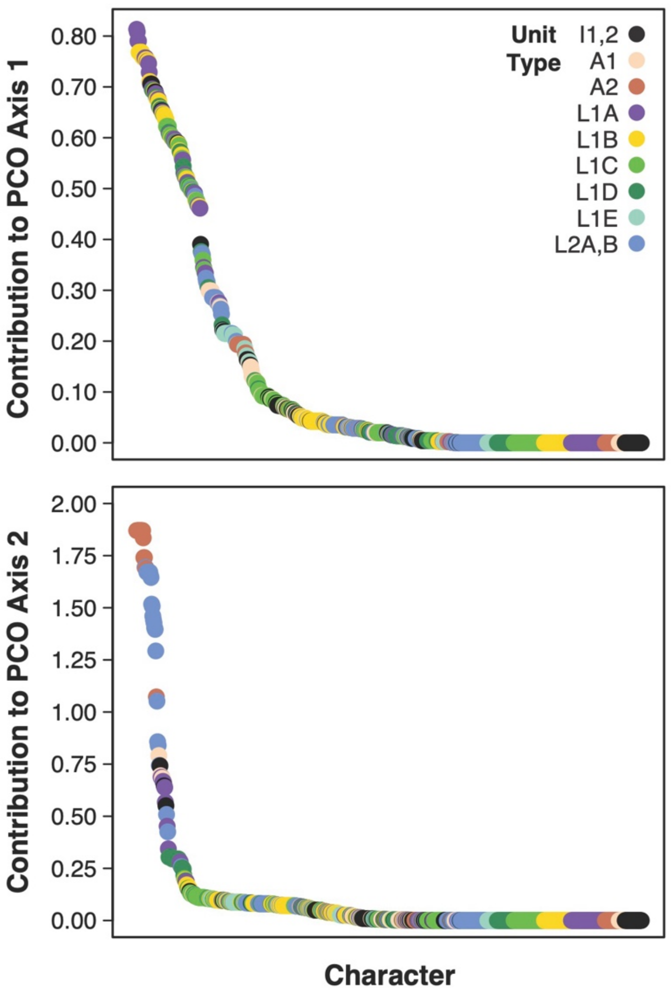

Figure S2. Total analysis Principal Coordinate (PCO) axes character associations for seed-producing structures. Characters are colored by general structural unit category for visualization. The strongest associations with PCO Axis 1 occur in L1A, L1B, and L1D organs, while those of PCO Axis 2 occur in A2, A1, and L2A organs. 'Contributions' are based on relative R-squared values between character data and PCO axis scores; see main text for explanation.

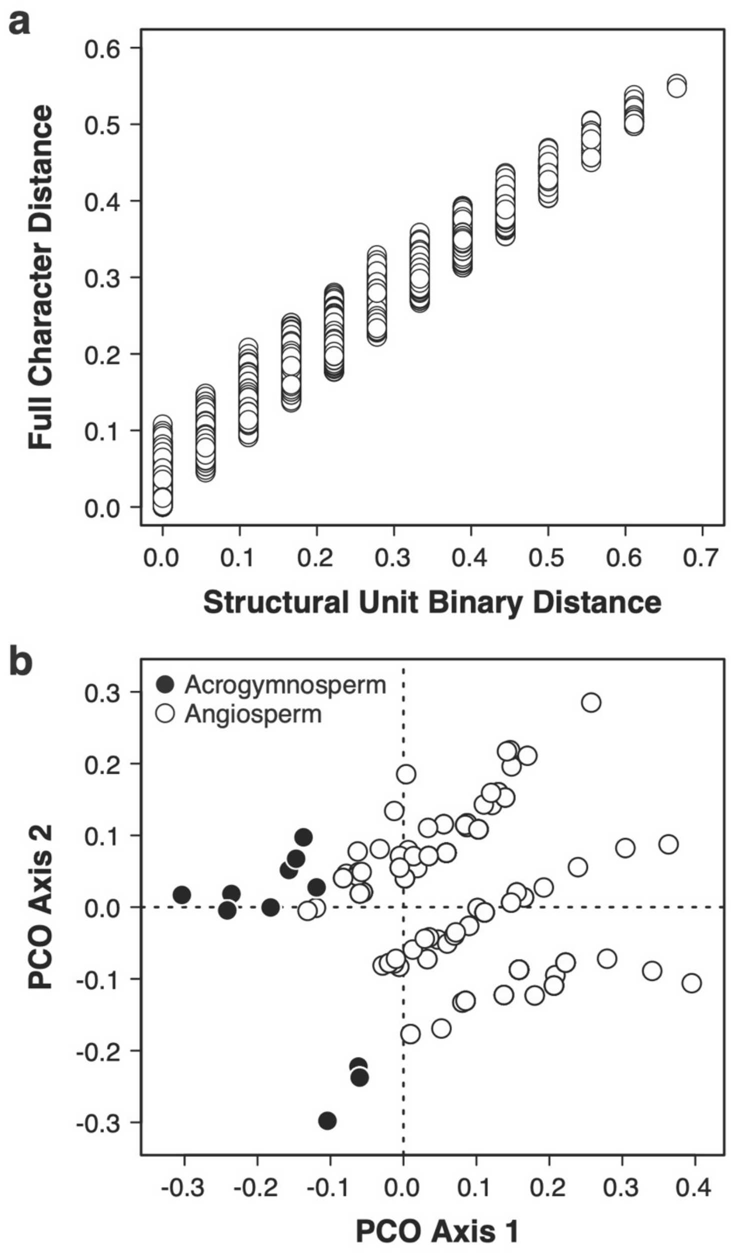

Figure S3. Results of analyses using binary presence/absence characters for structural units in seed-producing structures (a) Comparison of pairwise distances between a total analysis of seed-producing structures using full character data and one where structural unit presence/absence was treated as a binary character. For the latter analysis, all character data for each structural unit type was collapsed into a single presence/absence character. (b) Principal coordinate (PCO) ordination of distances from the structural unit presence/absence total analysis. Points correspond to structural unit combinations observed in real gymnosperm and angiosperm taxa.

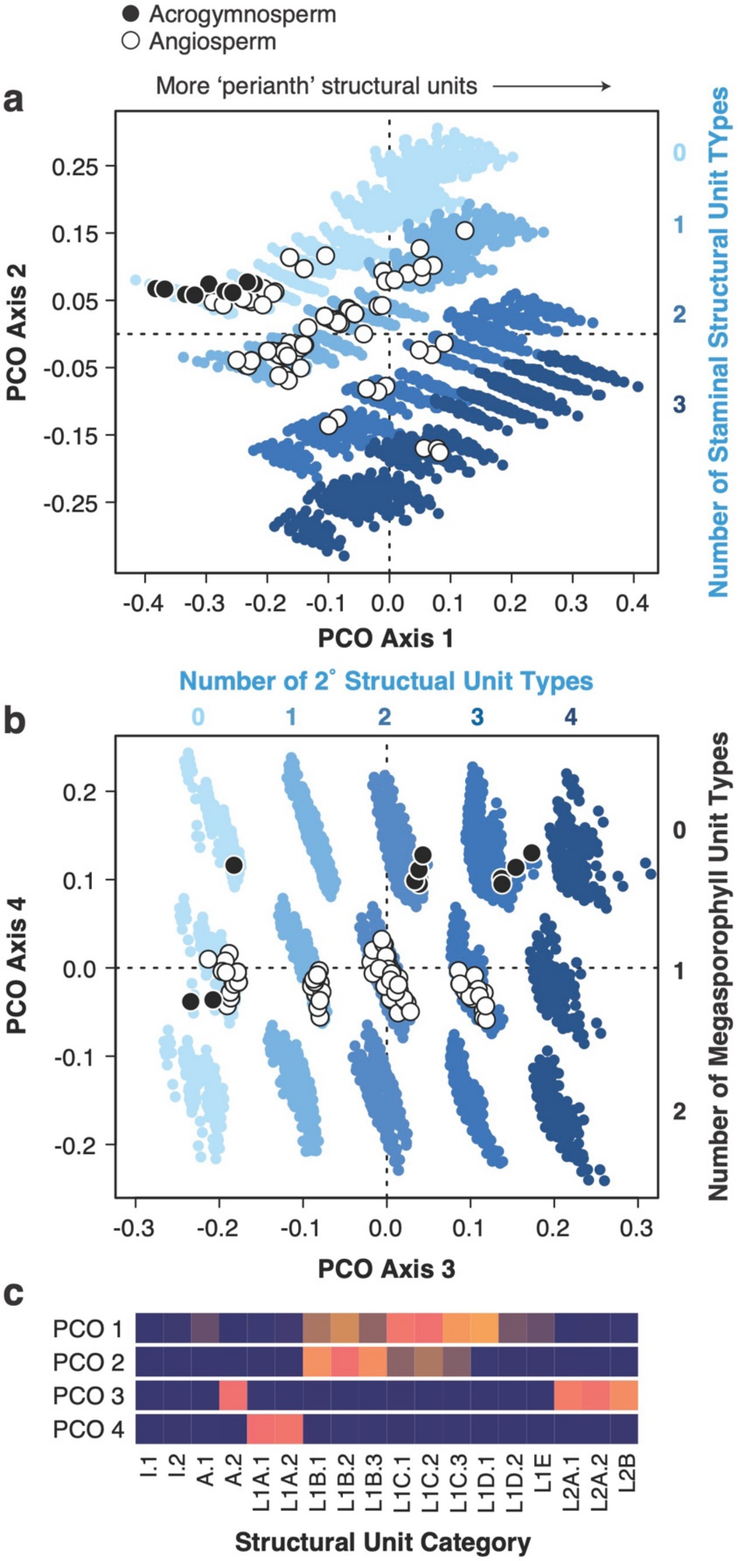

Figure S4. Morphospace ordinations of possible organ combinations among seed-producing structures. Ordinations are based on distances calculated from a version of the total analysis where structural unit presence/absence was treated as a binary character. (a) Principal Coordinates Analysis (PCO) Axes 1 and 2. Blue points represent simulated structural unit combinations with different hues representing different numbers of units; real combinations shown by black (acrogymnosperm) and white (angiosperm) points. Clustering on PCO Axis 2 is due to its relationship to number of staminal (L1B) units. (b) PCO Axes 3 and 4; clustering due to association of Axis 3 with second-order structural units (A2, L2A) and Axis 4 with megasporophylls (L1A). (c) heatmap showing associations between PCO axes and individual structural units, with blue indicating no association and red strong association. Associations based on R-squared values between structural units and PCO Axes in linear regression models; see main text for further explanation.

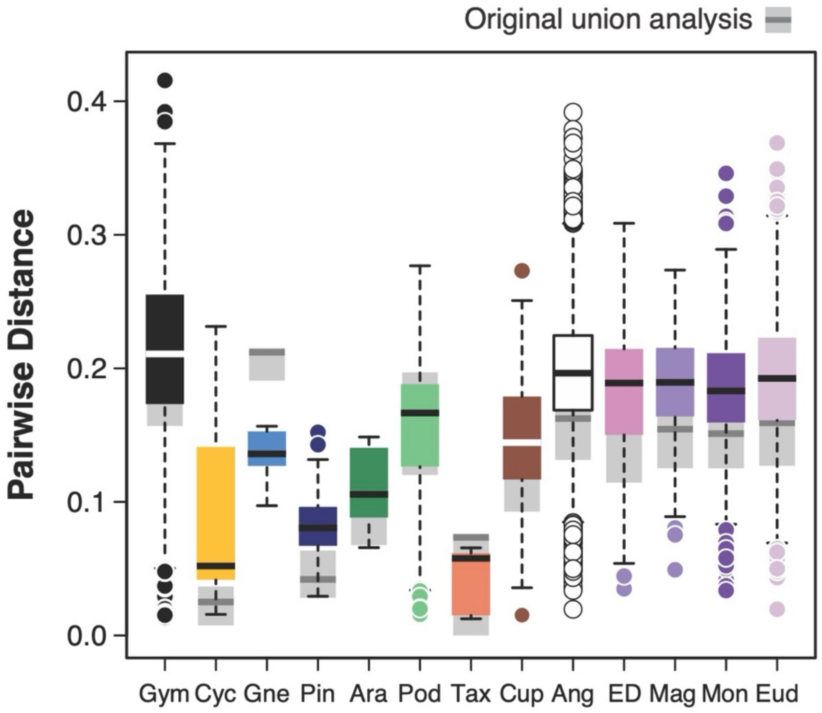

Figure S5. Comparison between morphological distances from original union analysis of seed-producing structures and those based on the 'classical' metric. In the latter, characters were not nested for analysis and non-applicable characters were removed from distance calculations. Boxplots show median (thick line), interquartile range (IQR; boxes), 1.5 times IQR (whiskers), and outliers. Gray boxes indicate median and IQR from the original union analysis. Ang = all angiosperms, Ara = Araucariaceae, Cup = Cupressaceae, Cyc = cycads, ED = 'early-diverging' angiosperms including ANA-grade, Ceratophyllum, and Chloranthaceae, Eud = eudicots, Gne = Gnetales, Gym = all gymnosperms, Mag = magnoliids, Mon = monocots, Pin = Pinaceae, Pod = Podocarpaceae, Tax = Taxaceae.

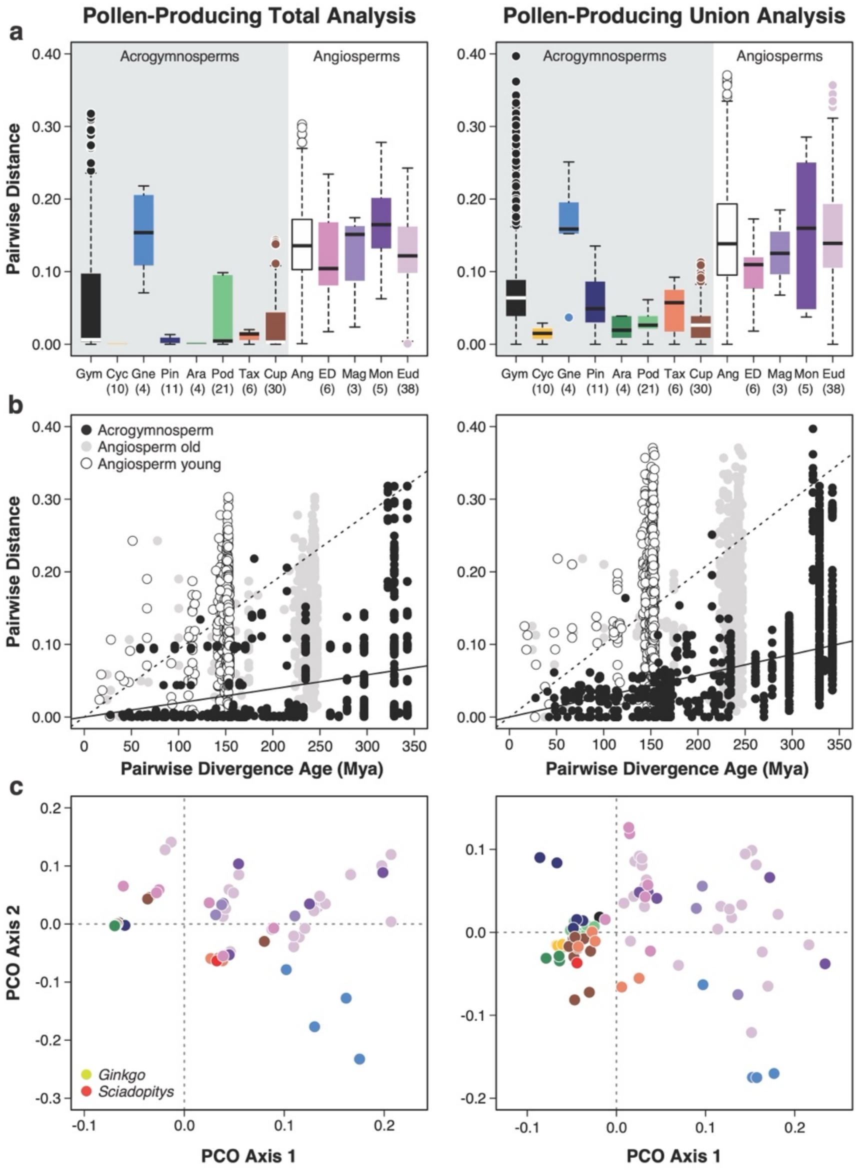

Figure S6. Pairwise morphological distances and morphospace occupation patterns in pollen-producing structures. (a) Within-group distances for acrogymnosperms and angiosperms in total and union analyses. Boxplots show median (thick line), interquartile range (boxes), 1.5 times interquartile range (whiskers), and outliers. Numbers below group names indicate number of taxa in each group. (b) Pairwise distances versus estimated pairwise divergence ages for acrogymnosperm and angiosperm taxa in total and union analyses. Two angiosperm ages represent alternative age calibrations; we favor young because they better align with the fossil record. Lines indicate best fit regression slopes for acrogymnosperms (solid) and angiosperms (dashed). (c) Principal Coordinate Analysis (PCO) Axes 1 and 2 for total and union analyses. Colors correspond to groups in (a,d) but with the addition of two monotypic acrogymnosperms (Ginkgo, Sciadopitys). Ang = all angiosperms, Ara = Araucariaceae, Cup = Cupressaceae, Cyc = cycads, ED = early-diverging angiosperms, including ANA-grade, Ceratophyllum, and Chloranthaceae, Eud = eudicots, Gne = Gnetales, Gym = all gymnosperms, Mag = magnoliids, Mon = monocots, Pin = Pinaceae, Pod = Podocarpaceae, Tax = Taxaceae.

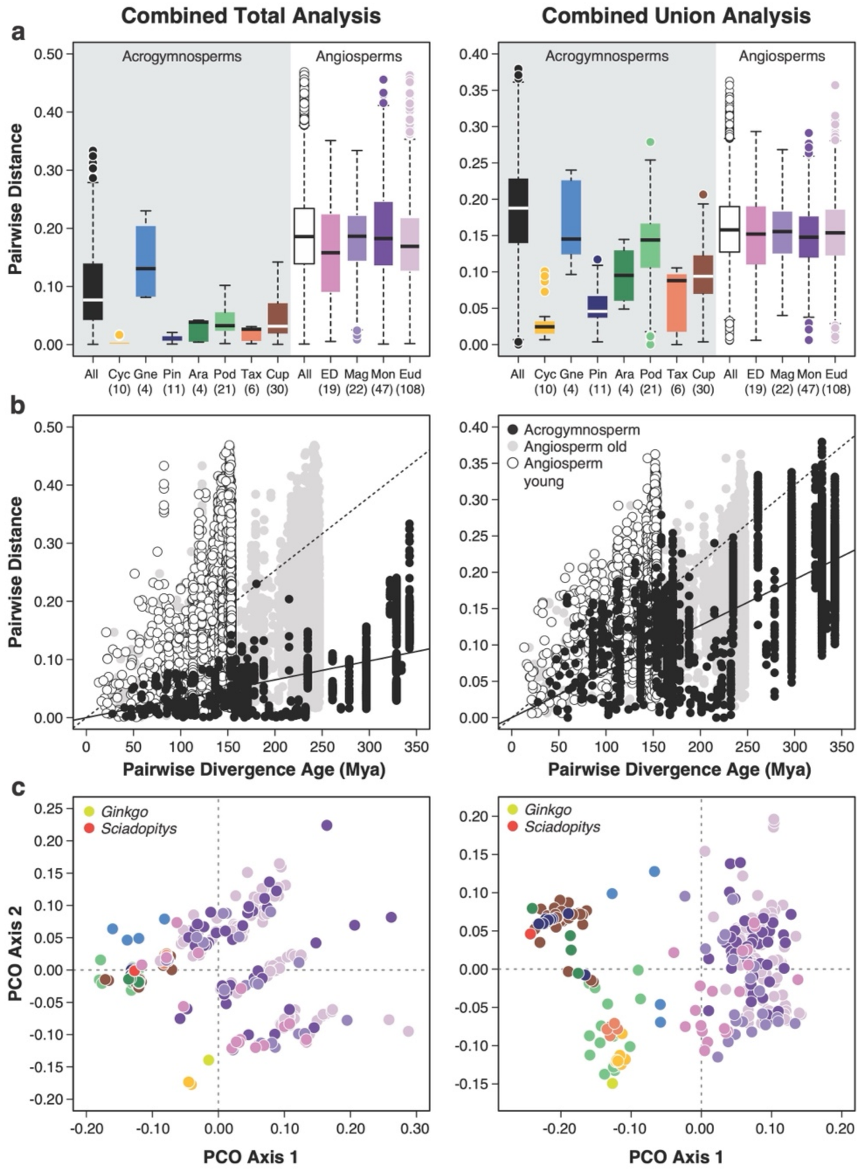

Figure. S7 Pairwise morphological distances and morphospace occupation patterns in combined structures. (a) Within-group distances for acrogymnosperms and angiosperms in total and union analyses. Boxplots show median (thick line), interquartile range (boxes), 1.5 times interquartile range (whiskers), and outliers. Numbers below group names indicate number of taxa in each group. (b) Pairwise distances versus estimated pairwise divergence ages for acrogymnosperm and angiosperm taxa in total and union analyses. Two angiosperm ages represent alternative age calibrations; we favor young because they better align with the fossil record. Lines indicate best fit regression slopes for acrogymnosperms (solid) and angiosperms (dashed). (c) Principal Coordinate Analysis (PCO) Axes 1 and 2 for total and union analyses. Colors correspond to groups in (a,d) but with the addition of two monotypic acrogymnosperms (Ginkgo, Sciadopitys). Ang = all angiosperms, Ara = Araucariaceae, Cup = Cupressaceae, Cyc = cycads, ED = early-diverging angiosperms, including ANA-grade, Ceratophyllum, and Chloranthaceae, Eud = eudicots, Gne = Gnetales, Gym = all gymnosperms, Mag = magnoliids, Mon = monocots, Pin = Pinaceae, Pod = Podocarpaceae, Tax = Taxaceae.

Table S1. Union analysis Principal Coordinate (PCO) axes character associations for seed-producing structures. Character numbers correspond to those listed in Supporting Notes below. 'Contributions' are based on relative R-squared values between character data and PCO axis scores; see main text for explanation.

Character	PCO Axis 1 Contribution	PCO Axis 2 Contribution
1.1.1	4.92	0.23
1.1.2	6.18	1.01
1.2	0.77	3.17
1.3	0.00	0.00
1.4.1	7.07	0.92
1.4.2	8.05	1.16
1.5	7.64	1.07
1.6	0.93	0.06
2	0.67	2.27
3	1.85	1.19
4	7.97	0.02
5	9.74	1.65
6	0.70	0.90
7	0.66	0.05
8	0.98	5.96
9	0.71	7.30
10	0.78	0.79
11	6.18	1.71
12	1.08	0.47
13	0.02	0.11
14	0.13	27.04
15.1	3.80	14.79
15.2	2.45	8.22
16.1	0.10	0.09
16.2	0.24	0.39
16.3	0.02	0.56
16.4	3.78	0.88
16.5	0.05	0.35
16.6	0.00	0.95
16.7	0.23	0.54
17.1	0.00	0.52
17.2	0.71	0.03
17.3	0.59	1.20
17.4	0.47	2.26
17.5	1.92	1.45
17.6	0.63	0.28
17.7	0.51	0.06
17.8.1	0.80	1.53
17.8.2	0.65	0.26
18.1	9.64	0.91
18.2	1.80	1.95
18.3	0.99	3.62
18.4	1.80	1.24
19.1	0.38	0.80
19.2	0.02	0.00
19.3	0.26	0.01
19.4	1.13	0.00

Supplementary Notes

Discrete Character List

The full list of characters and character states are provided below, in some cases with additional explanatory text. Nested character sets are indicated within { }. X represents a specific structural unit designation: A.1, A.2, L1A, L1B.1, etc. Character and state numbers follow those in the raw data provided on Dryad (DOI: 10.5061/dryad.qv9s4mwsw)

X.1. Reproductive organs (pollen sac or nucellus)

{1. Dehiscence

{1. Dehiscence type

1. Longitudinal
2. Transverse
3. Poricidal/restricted
4. Valvate

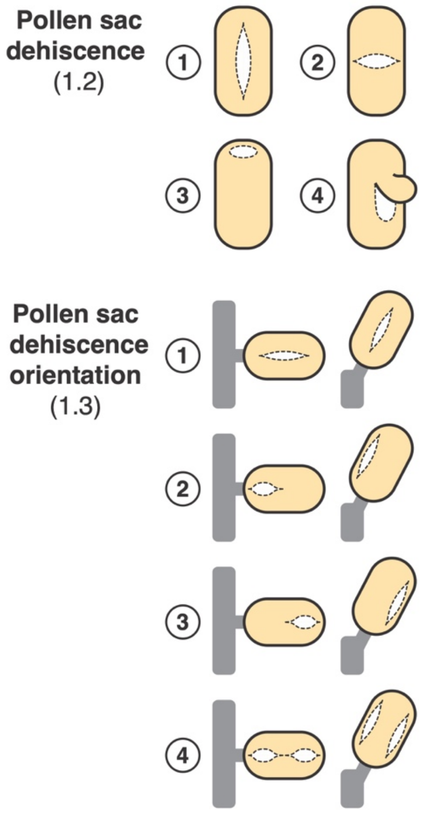

2. Dehiscence orientation

1. Latrorse
2. Introrse
3. Extrorse
4. Multiple orientations}

2. Chamber in organ wall

1. no
2. yes, from tissue degeneration
3. yes, from developed chamber

3. Accessory reproductive organ wall elements

1. none
2. tube
3. funnel
4. multiple types

4. Reproductive organ fusion

{1. Number of reproductive organs in fused structure (e.g., synangium)

1. 2
2. 3
3. 4
4. 5+

2. Organ attachment within synangium
    1. Point attachment
    2. Fusion across length}

5. Number of reproductive organs/synangium units borne on supporting organ
    1. 1
    2. 2
    3. 3-10
    4. 11-50
    5. 50+

6. Reproductive organ attachment to subtending organ
    1. Point attachment
    2. Partially fused along length or surface
    3. Fused over full surface}

Structural Unit Arrangement

X.2. Unit envelopes reproductive organ

1. No
2. Partially (>75%, <100%)
3. Fully

*Character refers to units that enclose reproductive organs directly with no intervening units such as integuments, carpels, etc.

X.3. Unit envelopes other unit(s)

1. No
2. Partially (>75%, <100%)
3. Fully}

X.4. Unit attached to other lateral unit

1. No
2. Yes

*Character distinguishes units that are borne on other lateral units (e.g., stamens born on petals) from those borne directly on an axial system.

X.5. Unit fused to itself

1. No
2. Yes

*Character refers to postgenital fusion between one region of a structural unit and another region of the same unit. Among extant taxa, variation in this character is restricted to angiosperm carpels, which may be either fully sealed or not.

X.6. Unit fused to another element of the same unit type

1. No
2. Partially (25-50% along unit length)
3. Mostly (51-75% along unit length)
4. Fully (> 75% along unit length)

*Character refers to fusion between different elements of the same unit type, either through postgenital or congenital fusion. We do not score units attached or fused only at the base as fused. Fusion along an edge, as can occur among carpels in some syncarpous ovaries, is treated as a case of partial fusion. Fused integuments are also scored using this character.

X.7. Unit fused to a different unit type

1. No
2. Partially (25-50% along unit length)
3. Mostly (51-75% along unit length)
4. Fully (> 75% along unit length)}

*Character refers to fusion between different structural unit types, either through postgenital or congenital fusion. We do not score units attached or fused only at the base as fused. Unit fusion along an edge, as can occur among carpels in some syncarpous ovaries, is treated as a case of partial fusion.

X.8 Unit adpressed along length to same unit type over ontogeny

1. No (<25% surface covered)
2. Yes, mostly exposed (25-50% surface exposed)
3. Yes, mostly covered (<50% surface exposed)

X.9. Unit adpressed along length to different unit type(s) over ontogeny

1. No (<25% surface covered)
2. Yes, mostly exposed (25-50% surface exposed)
3. Yes, mostly covered (<50% surface exposed)}

*Adpression refers to consistent physical contact between unfused units following bud break and prior to pollen or seed release. For pollen cones and staminal elements, this interval may be brief, but the character is meant to describe whether a unit grows and develops for a substantial part of its ontogeny in close contact with other units. Note that if adpressed units differ substantially in size, it is possible for the shorter unit to be adpressed along its length while the unit organ is not; e.g., a small calyx adhering to a larger fruit.

X.10. Number of units associated with subtending or unit-bearing structure

1. 1
2. 2
3. 3-10
4. 11-50
5. 50+

*Character refers to how many times the structural unit type is reiterated on whatever structure bears it; this could include lateral units like carpels or cone scales but also includes reiterated axes that comprise inflorescences.

X.11. Unit repetition arrangement

1. Indeterminate
2. Non-whorled
3. Whorled

*Here we have reduced various types of arrangement to simply whorled versus non-whorled. Non-whorled includes helical, decussate, and planar arrangements.

X.12. Number of structural units variable within individuals

1. No
2. Yes

Structural Unit Morphology

X.13. Unit branching

1. none
2. forking
3. pinnate

X.14. Unit develops fleshy/succulent tissues

1. No
2. Yes, over length
3. Yes, in part

X.15. Wing elements

{1. Wing development

1. Rudimentary (maximum wing height < 25% seed length)
2. Well-developed

2. Wing number

1. Continuous band
2. Multiple separate wings}

X.16. Scale elements

{1. Shape
1. Linear/oblong/lanceolate
2. Deltoid/cordate
3. Ovate/elliptical
4. Cuneate/flabellate
5. Reniform
6. Irregular

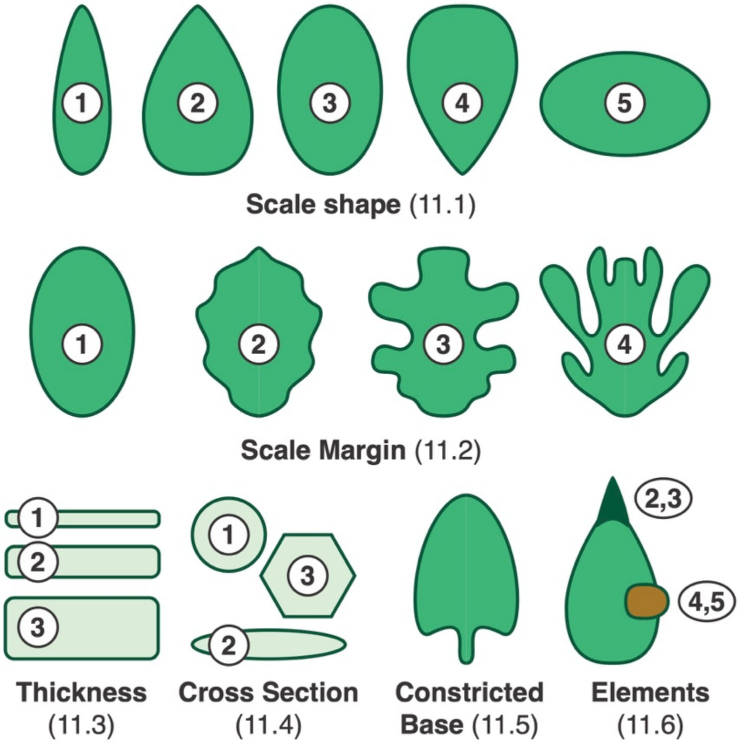

2. Margin
1. Entire
2. Shallow lobes (<20% length)
3. Lobed (20-50% length)
4. Deeply lobed (>50% length)

3. Thickness
1. Thin (<10% length)
2.Intermediate (10-30% length)
3. Thick (>30% length)

*Character is scored at unit maturity.

4. Cross section
1. Terete
2. Elliptical
3. Polyhedral

5. Constricted base
1. No
2. Yes

6. Additional elements
1. None
2. Small marginal element (<20% scale length)
3. Large marginal element (>20% scale length)
4. Flush umbo element (not appreciably raised)
5. Small umbo element (<20% scale length)
6. Large umbo element (>20% scale length)

*These features refer to addition projections on scale elements beyond those resulting from undulating margins. Marginal elements emerge from scale margins, while umbo elements

emerge from abaxial or adaxial scale surfaces. These features include many types of spines or prickles, although they may include thin sheets as well; for example, we typically treat coronal elements emerging from petals as umbo elements.

7. Scale asymmetry
    1. No
    2. Yes}

*Character refers to scale elements that are not bilaterally symmetric; for example, keel or wing petals in many Fabaceae flowers.

X.17. Ring elements

{1. Ring element complete
    1. No
    2. Yes

2. Ring element cup
    1. No
    2. Yes, shallow cup
    3. Yes, deep cup

*A shallow cup points outwards and has a low aspect, while a deep cup has straight or incurved walls. Envelopes, such as carpels, are typically scored as deep cups, although they may also be scored as tubes depending on their morphology.

3. Ring element tube/cylinder
    1. No
    2. Yes, short tube
    3. Yes, long tube

*A long tube is defined as having a length twice or more the diameter.

4. Ring element thickness
    1. Thin (<10% diameter)
    2. Intermediate (10-20% diameter)
    3. Thick (>20% diameter)

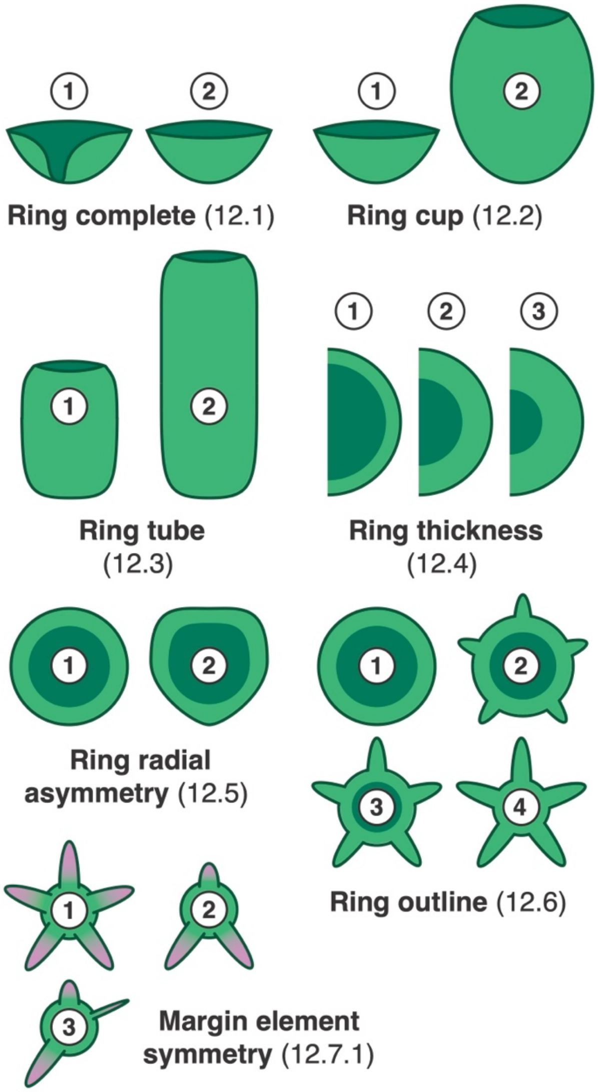

*For disk elements (rings without a central cavity), thickness is defined as half the diameter and is scored as state '3'. This character is scored at organ maturity.

5. Ring element radial asymmetry
    1. No
    2. Yes

*Character refers to the symmetry of the tube element itself rather than that of any marginal elements it may produce. Asymmetric rings are most commonly found in flattened or irregular ovaries.

6. Ring element outline
    1. Entire
    2. Shallow lobed (<20% ring radius)
    3. Lobed (20-50% ring radius)
    4. Deeply lobed (>50% ring radius)

*Ring radius refers to the total radius of the ring element, including any lobes.

7. Ring element attached to basal stalk element
    1. No
    2. Yes

*Character primarily distinguishes carpels/ovaries where the basal portion differentiates into a supporting stalk.

8. Ring margin elements

    {1. Margin element symmetry
        1. Radial
        2. Bilateral
        3. Irregular/asymmetrical

    2. Margin element number
        1. 1
        2. 2
        3. 3
        4. 4-5
        5. 6-10
        6. 11+}}

*Ring margin elements refer to projections at the margin of the ring element and are not present in all elements; as such they are a nested set of characters within ring element characters. These are most commonly scored in floral tubes with free petal or sepal lobes, which we score as

marginal elements attached to the floral tube. The relative length of these elements is accounted for by the extent of fusion among perianth organs (Character 4.2); for a more detailed treatment of how we treated the consequences of organ fusion, see notes following the character list.

X.18. Pollen reception elements

{1. Reception area on ovule
1. no
2. yes

2. Reception area projection
1. Absent
2. Short (< ovule/ovary length)
3. Thin elongate (>ovule/ovary length)
4. Columnar elongate
5. Laminar elongate
6. Variable

*Character refers to any elevated morphological element that receives pollen directly or via supporting a pollination drop.

3. Reception area morphology
1. No specialized region
2. Small pad/patch
3. Crest/linear element
4. Small flap/laminar element
5. Large flap/laminar element
6. Complex surface

4. Number of reception areas
1. Single from single base
2. Single from multiple bases
3. Multiple from single base
4. Multiple from multiple bases}

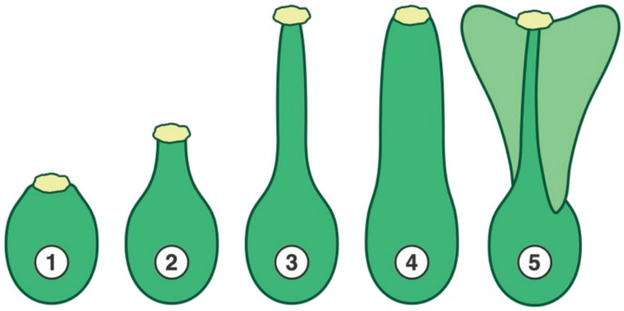

Style (13.1)

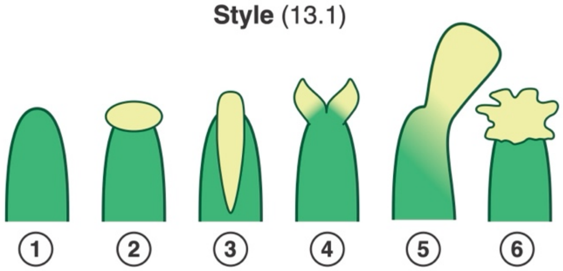

Reception Area Morphology (13.2)

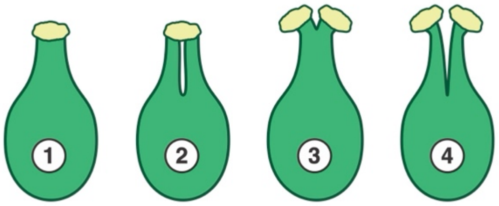

Reception Area Morphology (13.3)

X.19. Organ surface features

{1. Spines
1. No
2. Yes

2. Verrucae
1. No

2. Yes

3. Ridges
    1. No
    2. Yes

4. Glands
    1. No
    2. Yes}

*Because these are minor morphological components, these characters are nested in all analyses. These characters refer to either minor surface features on structural units or to various types of glands, which may include nectaries.

Discrete character scoring approach

We generally considered the entire functional ontogeny of a structural unit when scoring characters. For example, we scored scale thickness and fleshiness in conifer cone scales at maturity, while seed micropylar characters were scored at pollination. In angiosperms, carpel shape and arrangement characters were scored at fruit maturity while pollen reception characters were scored at pollination. For structural units with short ontogenies, such as stamens or petals, we scored them at anthesis unless they continued to mature with fruits. For characters relating to physical interactions, we scored these character as regards the majority of development. For example, adpression here refers to consistent physical contact between unfused units following bud break and prior to pollen/seed release rather than transient contact during expansion or growth. Often this means structural units shape is clearly affected by neighboring units, as for example in polygonal scales. Although the ontogeny of pollen cones and stamen structural units may be brief, characters are meant to describe whether structural units grow and develop for a substantial part of their ontogeny in close contact with others. Note that if adpressed organs differ substantially in size, it is possible for the shorter organ to be adpressed along its length while the longer organ is not; e.g., a small calyx adhering to a larger fruit.

Fusion is another potentially complicating factor in character scoring, because it can create additional structural elements. In general, we treated fused structures as we would any other structural unit. In the case of corolla or calyx tubes, for example, we considered the fused portion as a ring element and free petal/sepal lobes (if present) as individual scale elements that also represent marginal elements of the ring. When considering the number of reiterated elements in fused structures, we generally scored a fused structural unit as solitary, even if it is clearly composed of multiple elements. The only exception to this approach was for syncarpous ovaries, where we scored structural unit number as the total number of carpels (or locules) present. We scored these structures differently from other fused structural units because they retain an internal structure (in most cases) that clearly delineates the originally separate organs. In terms of other aspects of their morphology and arrangement, such as overall shape and

packing, we scored syncarpous ovaries as a single contiguous structural unit. In cases where fusion has been postulated but is not definitively known to have occurred, such as the fusion of bracts to form the 'arils' in Taxaceae seed cones, we did not score the organ as consisting of fused structures but rather as a single structure.

Angiosperm hypanthia and inferior ovaries also represent challenges for consistently scoring characters. We generally treated inferior ovaries as carpels to which other floral organs were attached. We did not consider inferior carpels to be fused into a calyx or other structural element unless these were clearly separate and identifiable structures, as in apples for example. For hypanthia, we treated the fused portion of the cup as being formed by sepal (L1D.1) elements, with stamens and petals (if present) regarded attached to the fused portion rather than composing the fused portion.

Structural unit categorization in the total analysis

To assign structural unit identities for our analyses, we first classified component parts of reproductive structures as integumentary (I), axial (A), and lateral (L).

We assigned ovule organs to either I.1 or I.2 subtypes, where I.1 is the integument and I.2 describes ovule-derived aril tissues. The latter would not normally be considered an integument, but for the purposes of this analysis we considered them to be comparable. We did not treat inner and outer integuments in angiosperms as separate organs, but rather as a single organ whose number corresponds to how many ovules are borne in an individual carpel or locule. We chose this approach because we felt that the number of ovules was a more important trait than the number of integuments.

We scored two types of axial organs; first-order axes A1, which were defined here as those that either directly bear pollen sacs/ovules or produce the corresponding lateral organs that bear them, and second-order axes A2, which are axes that bear first-order axes (or their derivatives). Although many angiosperm inflorescences produce higher orders of branching, we did not score them here for two reasons: 1) doing so would likely result in a morphospace dominated by differences in branching order among taxa, and 2) inflorescence axis elements are typically morphologically similar if not identical to second-order inflorescence axes. In some cases, axial components could not be directly observed but were inferred based on the presence of lateral organs; for example, a solitary, sessile flower may not show an obvious axis. In such cases, we scored axial structural units as present but as lacking any specific morphological features. We also scored inflorescences based on one individual or example. This ignores the large amount of variability that can exist within angiosperm taxa in terms of inflorescence size and degree of reiteration, but the primary focus of this study is on the reproductive morphology of the major functional units rather than inflorescence diversity/variability.

For lateral organs borne on first-order axes, we assigned them to one of several L1 categories. L1A and L1B units represent first-order lateral organs that bear ovules and pollen sacs,

respectively; this corresponds to megasporophylls/carpels and microsporophylls/stamens. L1A units were further subdivided into L1A.1, the seed-bearing megasporophyll or carpel, and L1A.2, a category for sterilized organs like pistillodes. LIB units were further subdivided into L1B.1, the fertile stamens or sporophylls, and L1B.2 and L1B.3, which are categories for staminodes. Additional sterile lateral organs were then placed in one or more categories of 'perianth' organs following a hierarchical scheme. We first placed any additional L1 units beyond L1A and L1B into the L1D category, which corresponds to perianth in the broadest sense and includes bracts, tepals, and sepals (in taxa with further differentiated perianth). If distinct petals/corolla organs were also present in addition to L1D units, we placed these in the L1C category. This category also includes petaloid tepals. Both L1C and L1D categories also include the potential for multiple sub-organs in order to accommodate differentiated petals and sepal elements. For example, Fabaceae flowers often have petals that are differentiated into morphologically distinct types (e.g., keel and wings). We added additional the organs to these subcategories in no particular order; for example, if a taxon produced two distinct types of staminodes, these were sequentially added as L1B.2 and L1B.3 units. If accessory bracts or bracteoles were present on first-order axes proximal to L1D units, these were placed in the L1E category. Although the L1 categories were primarily created to accommodate the typical arrangement of angiosperm flowers, they are applicable to gymnosperms as well. For example, some Cupressaceae pollen cones (e.g., *Cunninghamia*, *Taiwania*) produce basal bracts or bracteoles that we score as L1D units because they represent undifferentiated 'perianth'.

For organs borne on second-order axes, we scored two types: L2A and L2B. L2A units include bracts that subtend first-order axes (e.g., flower subtending bracts) as well as bracts that subtend lateral inflorescence branches. If these kinds of bracts were morphologically identical, they were scored as one organ type. If instead partial inflorescence bracts exhibited consistent differences in size or morphology, we scored them as an L2A subcategory (L2A.2). Lastly, the L2B category corresponds to bract or bud scales that subtend an entire reproductive structure or inflorescence, such as spathes in many monocots. For individual solitary flowers subtended by a bract, this bract was treated as an L2A.1 organ for consistency with other taxa.

It is important to emphasize that these organ categories are not homologous and that they are intended only to provide a framework of functionally and architecturally analogous organs in which to compare taxa. The deeper homologies of these units are not considered in our categorization. For example, the carpel has been interpreted as a bract subtending a fertile axis by some authors (e.g., Shi et al., 2021), in which case it should more properly be scored as a L2B organ. Although such hypotheses can readily be accomodated by modifying our current system to add more subcategories of L2B organs, assessing the true origins of plant organs is not the goal of this study.

Structural Unit Dependencies

Based on the concepts discussed in the preceding section, we used the following rules to calculate different possible organ combinations for the theoretical total analysis.

1. All taxa have I.1 in combined/ovulate analysis
2. If no L1A1: no L1A.2
3. If no L1B1: no L1B.2, L1B.3
4. If no L1B2: no L1B.3
5. If no L1C.1: no L1C.2, L1C.3
6. If no L1C.2: no L1C.3
7. If no L1D1: no L1C, L1D2, L1E
8. If no L1C.1: no L1C.2, L1C.3
9. If no L1C.2: no L1C.3
10. If no A2: no L2A.2, L2B
11. If no L2A1: no L2A.2

Structural unit categorization for groups in the total analysis

Cycads and *Ginkgo* - We treat these groups as exhibiting only A1 axes for both pollen and seed cones. These axes correspond to the pollen/seed cone axis (or in the case of *Cycas* seed cones, the main trunk of the plant). In both groups, pollen-sac bearing organs are considered L1B microsporophylls, while ovule/seed bearing organs in cycads are categorized as L1A megasporophylls. In *Ginkgo*, the seed supporting pedicel is scored as an axial organ while the seed 'collar' is scored as a reduced L1A megasporophyll.

Gnetales - We treat Gnetales reproductive structures as compound cones. We treat Gnetales seeds as borne on highly reduced A1 axes and surrounded by one or more envelopes consisting of modified bracts. We treat these envelope organs as L1D 'perianth' following our general protocol. In *Ephedra* and *Gnetum*, we treat ovulate A1 axes as borne on A2 axes bearing various L2A bracts (collar bracts in *Gnetum*, fleshy or dry bracts in *Ephedra*). In *Welwitchia* ovulate cones, we score additional L1E lateral bracteole organs proximal to the winged seed envelope, with the entire axial dwarf shoot subtended by L2A.1 cone scale bracts. The pedunculate *Welwitschia* seed cones are in turn subtended by L2A.2 bracts.

Staminate cones in Gnetales are complex. We treat the antherophores of *Ephedra* and *Gnetum* as A1 fertile axes that bear pollen sacs and L1D.1 perianth organs at their base. We also score additional L1D.2 perianth organs in *Gnetum* and some *Ephedra* staminate cones in order to accommodate the envelopes around the sterile nectar-producing ovules. *Gnetum* and *Ephedra* staminate cones both produce multiple antherophore units subtended by L2A bracts, although in *Gnetum* the antherophores occur in whorls subtended by a single ring-like collar bract. We consider the antherophores of *Welwitschia* to represent fused L1B microsporophyll organs rather than an A1 axis, although these organs have been interpreted as a partially fused shoot axis (Mundry and Stutzel, 2004). Here we score the lateral dwarf shoots of *Welwitschia* pollen cones as A1 units, which bear the sterile ovules, antherophores, and lateral L1E bracteoles. The pollen cones of *Welwitchia* are otherwise similar in structure and organography to the seed cones.

Conifers - Following standard interpretations of the conifer seed cone, we generally considered

them to be compound structures. Organs regarded as ovuliferous scales or derivatives (like epimatia), which are found among some early-diverging Cupressaceae (*Cunninghamia* and Taxodioideae), Pinaceae, and Sciadopityaceae, are therefore considered modified A1 axial units. The bracts subtending ovuliferous scales, which sometimes provide the basic structure of the cone, are then considered L2A.1 organs borne on the main cone A2 axis.

Derived Cupressaceae seed cones are generally interpreted as lacking an ovuliferous scale, although these organs are often considered to be present but entirely fused with subtending bracts (Schulz and Stutzel, 2007). Here we do not infer their existence; we consider A1 axial organs to be absent if there is no clear evidence of an axillary structure. This interpretation includes taxodiaceous Cupressaceae for which ovules are borne directly on the bracts (e.g., *Taiwania*, *Metasequoia*) and Cupressaceae sensu stricto where ovules are axillary (e.g., *Thuja*, *Cupressus*). In the latter cases, ovules/seeds are scored as borne on the A2 cone axis directly, along with the L2A bracts which comprise the seed cone.

Podocarpaceae includes a wide diversity of seed cone morphologies, although most are interpretable as modified seed-bearing ovuliferous scales (called epimatia in this group) subtended by L2A bracts. These units may occur singly or in multiples, and additional sterile L2A bracts may be present as well, together forming a fleshy receptacle. In some taxa, however, the ovuliferous scale has apparently been lost (e.g., *Pherosphaera*); these cones are scored in the same manner as derived Cupressaceae seed cones. *Phyllocladus* represents a special case where the fleshy organ surrounding the seed has traditionally been considered an 'aril' rather than an ovuliferous scale. Here we score it as an A1 ovuliferous scale organ based on its positional similarity to other taxa with epimatia.

The homologies of Taxaceae seed cones are perhaps the most contentious and obscure of all extant conifers. We generally consider the fleshy 'aril' to represent an L1D 'perianth' unit analogous to Gnetales seed envelopes. This categorization is based on the idea that the 'aril' forms from modified bracts (Dorken et al., 2019) that would be borne on a first-order fertile axis. We consider the rest of the seed cone axis in Taxaceae members, however, to be a second-order A2 axis bearing L2A bracts. This somewhat unusual interpretation is based on *Cephalotaxus*, the likely sister group to other Taxaceae, which produces pairs of 'aril'-covered seeds in an axillary position subtended by a bract. For the sake of consistency across the group, we therefore considered the 'aril'+seed units to represent the remains of a primary fertile axis that is either directly attached to a secondary A2 axis (most Taxaceae) or borne in an axillary arrangement around an A2 axis (*Cephalotaxus*). Other interpretations of Taxaceae homology could readily be implemented in our scoring scheme, however; for example, the 'aril' could be scored as an A1 ovuliferous scale, or the main cone axis in taxa other than *Cephalotaxus* could be scored as A1 axial organs.

Conifer pollen cones are generally regarded as first-order axes bearing microsporophylls, although some taxa produce compound pollen cones (e.g., some *Afrocarpus* and *Prumnopitys* species). Some Taxaceae pollen cones appear simple but likely represent compound axes based on the presence of subtending bracts below what would otherwise appear to be L1B

microsporophylls. Here we follow the organ interpretations of Schulz et al. (Shulz et al., 2014; Dorken and Nimsch, 2023) and consider all taxa except *Torreya* to produce compound cones. In *Pseudotaxus* and *Taxus*, we consider the pollen cone microsporophyll unit to consist of an A1 axis (the stalk of the microsporophyll) bearing a shield-like scutum (L1B unit) that represents the fused remains of two microsporophyll distal lamina.

Angiosperms - Angiosperm structural units are in some ways easier to categorize than acrogymnosperms because their basic homologies are generally agreed upon. In many particular cases, however, the loss or heavy modification of floral organs may make assignment difficult. In general, if flowers have become secondarily simplified and lost organ differentiation, we do not attempt to assess or maintain true identities; for example, if a flower shows only a single type of perianth element, we assign these organs to the L1D category regardless of whether they are developmentally derived from petals or sepals.

A full list of organ categories and assignments for all scored taxa and groups is available on Dryad (DOI: 10.5061/dryad.qv9s4mwsw)

References

Dörken VM, Nimsch H. 2023. Anomalous pollen cones in *Pseudotaxus chienii* (Taxaceae): a further support for the pseudanthial origin of the *Taxus* pollen cone. *Feddes Repertorium* 134:149-156.

Dörken VM, Nimsch H, Rudall PJ. 2019. Origin of the Taxaceae aril: evolutionary implications of seed-cone teratologies in *Pseudotaxus chienii*. *Annals of Botany* **123**: 133-143.

Mundry M, Stützel T. 2004. Morphogenesis of the reproductive shoots of *Welwitschia mirabilis* and *Ephedra distachya* (Gnetales), and its evolutionary implications. *Organisms Diversity & Evolution* **4**: 91-108.

Schulz, C, Klaus KV, Knopf P, Mundry M, Dörken V, Stützel T. 2014. Male cone evolution in conifers: not all that simple. *American Journal of Plant Sciences* **5**: 2842-2857.

Schulz C, Stützel T. 2007. Evolution of taxodiaceous Cupressaceae (Coniferopsida). *Organisms Diversity & Evolution* **7**:124-135.

Shi G, Herrera F, Herendeen PS, Clark EG, Crane PR. 2021. Mesozoic cupules and the origin of the angiosperm second integument. *Nature* **594**: 223-226.

Stull GW, Qu XJ, Parins-Fukuchi C, Yang YY, Yang JB, Yang ZY, Hu Y, Ma H, Soltis PS, Soltis DE, Li DZ. 2021. Gene duplications and phylogenomic conflict underlie major pulses of phenotypic evolution in gymnosperms. *Nature Plants* **7**: 1015-1025.

Zuntini AR, Carruthers T, Maurin O, Bailey PC, Leempoel K, Brewer GE, Epitawalage N, Françoso

E, Gallego-Paramo B, McGinnie C, Negrão R. 2024. Phylogenomics and the rise of the angiosperms. *Nature* **629**: 843-850.
